# Cardiometabolic Effects of Antipsychotics in Challenging Behavior and Neuropsychiatric Symptoms in Patients With Intellectual Disability

**DOI:** 10.7759/cureus.48088

**Published:** 2023-11-01

**Authors:** Tomoyuki Takano

**Affiliations:** 1 Department of Pediatrics, Biwako-Gakuen Medical and Welfare Center of Yasu, Yasu, JPN; 2 Department of Pediatrics, Shiga University of Medical Science, Otsu, JPN

**Keywords:** profound disability, cardiometabolic adverse effect, intellectual disability, neuropsychiatric symptom, challenging behavior, antipsychotics

## Abstract

Introduction

Despite the well-established association between antipsychotics and metabolic adverse effects in psychiatric disorders, the variable influence of long-term and off-label antipsychotic medication on behavioral disorders has not been investigated in detail.

Objective

To clarify the impact of antipsychotic use on challenging behavior (CB) and neuropsychiatric symptoms (NPS), we evaluated the clinical findings and cardiometabolic effects in individuals with intellectual disability (ID) hospitalized in our residential facility for profoundly disabled patients.

Patients and methods

A total of 130 individuals hospitalized in our residential facility were retrospectively investigated and divided into two groups - individuals with and without CB and NPS. Antipsychotics (first- and second-generation antipsychotics) are prescribed to all individuals in the CB and NPS-positive ID group. Conversely, antipsychotics were not prescribed to any individuals in the CB- and NPS-negative ID groups. To investigate the cardiometabolic effects of antipsychotics, the following metabolic parameters were measured: body weight (BW), total calorie intake per day, body mass index (BMI), blood pressure, serum lipid levels (total cholesterol, high-density lipoprotein cholesterol, and triglyceride), and corrected QT interval (QTc) of electrocardiogram (ECG). The etiology of disabilities, level of ID, severity of motor disability, and presence or absence of epilepsy were also examined.

Results

Two main results were derived: (1) Autism spectrum disorder was the most predominant etiology in individuals with CB and NPS who were prescribed antipsychotics, and (2) the mean values of metabolic parameters were all normal in both medicated and nonmedicated individuals, but the total calorie intake was significantly higher in individuals who were prescribed antipsychotics.

Conclusions

Our investigation demonstrated that in individuals with an adequate energy intake, most metabolic parameters can be maintained within normal limits. We suggest that body weight control is the primary step in avoiding the cardiometabolic adverse effects of antipsychotics in intellectually disabled individuals with CB and NPS.

## Introduction

Intellectual disability (ID) is a disorder with an onset during the developmental period that includes both intellectual and adaptive functioning deficits in conceptual, social, and practical domains [1]. The term "challenging behavior" (CB) has replaced several related terms, such as abnormal, aberrant, disordered, disturbed, dysfunctional, maladapted, and problem behaviors. These terms have previously been used to describe broad classes of behaviors shown by individuals with severe ID. They include aggression, destructiveness, self-injury, stereotyped mannerisms, and a range of other behaviors, which may be either harmful to the individual (e.g., eating inedible objects), challenging for carers and care staff (e.g., noncompliance, persistent screaming, disturbed sleep patterns, and overactivity), and/or objectionable to members of the public (e.g., regurgitation of food or smearing of feces over the body) [2]. In addition to CB, individuals with cognitive impairment are at high risk of developing various neuropsychiatric symptoms (NPS), such as apathy, depression, anxiety, delusions, hallucinations, agitation, and disinhibition [3]. CB and various NPS can seriously impede social interaction and participation in everyday activities, having a negative effect on not only health and well-being but also on the quality of life in most individuals with ID.

The basis of intervention to support individuals with ID who have behavioral and neuropsychiatric problems should be constructional, functional, socially valid, and ethical [2]. It is suggested that antipsychotics may be necessary when behavioral interventions cannot eliminate problem behaviors adequately or when behavior poses a risk to patients or their caregivers [4]. However, psychotropic drugs, in general, and antipsychotics, in particular, are overused in individuals with ID. They might often be prescribed for CB and various NPS rather than for diagnosed mental illness (i.e., off-label usage), which has been a concern for many years [5]. Despite the well-established association between antipsychotic use and significant metabolic and cardiac adverse effects in many psychiatric disorders [6], variable effects of long-term and off-label antipsychotic medication in individuals with behavioral and/or neuropsychiatric disorders have not been investigated in detail. To clarify the impact of antipsychotic use on CB and NPS, we evaluated the clinical findings and cardiometabolic effects in individuals with ID who were hospitalized in our residential facility for profoundly disabled patients.

## Materials and methods

Participants

This study was conducted from April 1, 2022, to March 31, 2023, at the residential facility hospital Biwako-Gakuen, in which medical and welfare treatment is provided to children and adults with intellectual and physical disabilities. Because of their profound disabilities, most individuals need to be hospitalized for their entire life. The total number of beds is 143, including 130 beds for lifelong admission and 13 beds for short-term admission. This study included all patients of lifelong admission (130 individuals) with intellectual and physical disabilities whose hospitalization period ranged from one to 58 years. This study was approved by the Ethics Committee of the Biwako-Gakuen Medical and Welfare Center of Yasu.

Definitions and instruments

ID was diagnosed based on the three domains of adaptive deficits as described in the Diagnostic and Statistical Manual of Mental Disorders, Fifth Edition (DSM-5) [1]: the conceptual, social, and practical domains.

CB was assessed with reference to the Behavior Problems Inventory-01 (BPI) [7], which is a 52-item respondent-based behavior rating instrument. The BPI consists of three subscales: self-injurious behavior (14 items), stereotyped behavior (24 items), and aggressive/destructive behavior (11 items). Self-injurious behavior is defined as behavior that can cause damage to the subject’s own body and occur repeatedly in an essentially unvarying manner (e.g., self-biting, head-hitting, and pica). Stereotyped behaviors are peculiar or inappropriate voluntary acts that occur habitually and repetitively (e.g., body movements, yelling, and finger movements). Aggressive or destructive behaviors are abusive, deliberate attacks against other individuals or objects (e.g., hitting, biting, and scratching). The professional caregivers in our residential hospital evaluated the frequency and severity of the particular behavior in a subject during the past two months on a 24-hour basis each day. Frequency was rated on a four-point scale of none, monthly, weekly, and daily, and severity was rated on a three-point scale of slight, moderate, and severe problems. The three subscales of CB were diagnosed when the frequency was more than monthly and the severity was more than a slight problem.

NPS was assessed with reference to the Neuropsychiatric Inventory (NPI), which was developed to assess psychopathology in dementia patients and can be applied in the evaluation of adults with ID [8]. It evaluates 12 neuropsychiatric disturbances that are common in dementia: delusions, hallucinations, agitation, dysphoria, anxiety, apathy, irritability, euphoria, disinhibition, aberrant motor behavior, night-time behavior disturbances, and appetite and eating abnormalities. The severity and frequency of each neuropsychiatric symptom are rated based on scripted questions administered to the patient’s caregiver [9]. In the present study, the frequency and severity of each symptom in a subject during the past two months were evaluated by the professional caregivers of our residential hospital on a 24-hour basis each day. Frequency was rated on a four-point scale of none, monthly, weekly, and daily, and severity was evaluated on a three-point scale of slight, moderate, and severe problems. CB was also evaluated. The diagnosis of NPS was made when the frequency was more than monthly and the severity was more than a slight problem.

Procedure

A total of 130 individuals hospitalized in our residential facility were retrospectively investigated and divided into two groups: individuals with CB and NPS (CB- and NPS-positive ID group) and individuals without CB and NPS (CB- and NPS-negative ID group). Antipsychotics (first- and second-generation antipsychotics) are prescribed to all individuals in the CB and NPS-positive ID group. Conversely, antipsychotics were not prescribed to any individuals in the CB- and NPS-negative ID groups.

The following items were collected from medical records: sex, age, hospitalization period, etiology of disabilities, level of ID, severity of motor disability, and presence or absence of epilepsy. The level of ID was evaluated by the intelligence quotient (IQ) using the New Edition K-type Developmental Test (one of the most current developmental tests adopted in Japan) and classified into four categories of disability: mild (IQ: 50-69), moderate (IQ: 35-49), severe (IQ: 20-34), and profound (IQ < 20). The severity of motor disability was evaluated and classified according to gross motor function into five levels: (1) run, (2) walk, (3) walk with assistance, (4) sit, and (5) bedridden.

To investigate the cardiometabolic effects of antipsychotic medication, the following metabolic parameters were measured: body weight (BW), total calorie intake per day, body mass index (BMI), blood pressure, serum lipid levels (total cholesterol, high-density lipoprotein cholesterol, and triglyceride), and corrected QT interval (QTc) of electrocardiogram (ECG). In individuals with CB and NPS-positive ID, sex, age, type of antipsychotics, medication period, and type of CB and NPS were examined.

Statistical analyses

Statistical significance was determined using Welch’s t-test and Fisher’s exact test. P-values of <0.05 were considered to indicate statistical significance. All analyses were performed using the Mac Statistical Analysis software program, version 3 (ESUMI Co. Ltd., Tokyo, Japan).

## Results

Clinical differences between ID with and without CB and NPS

Among a total of 130 individuals, 35 were classified into the CB- and NPS-positive ID group (26.9%; male/female, 18/17; mean age, 51.4 years [range: 19-72]; mean hospitalization period, 30.5 years [range: 1-57]), as shown in Table 1. Ninety-five individuals were classified into the CB- and NPS-negative ID group (73.1%; male/female, 59/36; mean age, 48.5 years [range: 12-76]; mean hospitalization period, 29.6 years [range: 1-58]). An analysis of the etiologies of ID revealed the following 10 disorders: chromosomal abnormalities, congenital malformation syndrome, malformation of cortical development, degenerative neurological disorders, neurocutaneous syndrome, epileptic encephalopathy, neurodevelopmental and psychiatric disorders, perinatal brain injury, central nervous system infection, and traumatic brain injury. The prevalence of neurodevelopmental and psychiatric disorders was significantly higher in the CB- and NPS-positive group (31.4%) than in the CB- and NPS-negative group (8.4%) (p < 0.001). Furthermore, autism spectrum disorder was the most predominant etiology among the evaluated neurodevelopmental and psychiatric disorders in individuals with CB and NPS (36.3%). Most individuals had a profound ID. The incidence of profound intellectual disability in the CB- and NPS-positive ID and CB- and NPS-negative ID groups did not differ to a statistically significant extent. Motor function more than walking with assistance (walking with assistance, walking, and running) was maintained in the CB- and NPS-positive ID groups (p = 0.03, 0.006, and 0.024, respectively); in contrast, most ID individuals in the CB- and NPS-negative ID groups had more severe motor disability, such as less than sitting and/or bedridden level. The prevalence of comorbid epilepsy in the CB- and NPS-positive ID and CB and NPS-negative ID groups did not differ to a statistically significant extent.

**Table 1:** Clinical differences between ID with and without CB and NPS CB: Challenging behavior; NPS: Neuropsychiatric symptom; ID: Intellectual disability; M/F: Male/female; SD: Standard deviation; BW: Body weight; BMI: Body mass index; QTc: Corrected QT interval; ns: Not significant. * All individuals are prescribed antipsychotics. ** Antipsychotics are not prescribed to any individual.

	ID with CB and NPS (*)	ID without CB and NPS (**)	P-value
Number of patients (M/F) (%)	35 (18/17) (26.9%)	95 (59/36) (73.1%)	0.271
Mean age, years (SD); range	51.4 (11.9); 19-72	48.5 (15.6); 12-76	0.259
Mean hospitalization period, years (SD); range	30.5 (18.1); 1-57	29.6 (19.5); 1-58	0.802
*Etiology of ID*			
Chromosomal abnormalities	0 (0.0%)	5 (5.3%)	0.166
Congenital malformation syndrome	3 (8.5%)	8 (8.4%)	0.978
Malformation of cortical development	1 (2.9%)	7 (7.4%)	0.342
Degenerative neurological disorders	1 (2.9%)	4 (4.2%)	0.721
Neurocutaneous syndrome	1 (2.9%)	4 (4.2%)	0.721
Epileptic encephalopathy	4 (11.4%)	10 (10.5%)	0.882
Neurodevelopmental and psychiatric disorders	11 (31.4%)	8 (8.4%)	<0.001
Autism spectrum disorder	4 (36.3%)	3 (37.5%)	ns
Unspecified neurodevelopmental disorder	3 (27.3%)	5 (62.5%)	ns
Schizophrenia	1 (9.1%)	0 (0.0%)	ns
Bipolar disorder	3 (27.3%)	0 (0.0%)	ns
Perinatal brain injury	11 (31.4%)	32 (33.7%)	0.808
Central nervous system infection	2 (5.7%)	11 (11.6%)	0.322
Traumatic brain injury	1 (2.9%)	6 (6.3%)	0.438
*Level of ID (IQ)*			
Mild (50-69)	1 (2.9%)	1 (1.1%)	0.458
Moderate (35-49)	2 (5.7%)	3 (3.1%)	0.501
Severe (20-34)	5 (14.3%)	7 (7.4%)	0.226
Profound (<20)	27 (77.1%)	84 (88.4%)	0.106
*Severity of motor disability*			
Bedridden	15 (42.9%)	51 (53.7%)	0.273
Sit	5 (14.3%)	35 (36.8%)	0.013
Walk with assistance	6 (17.1%)	5 (5.3%)	0.03
Walk	5 (14.3%)	2 (2.1%)	0.006
Run	4 (11.4%)	2 (2.1%)	0.024
*Epilepsy (%)*			
Positive	20 (57.1%)	60 (63.2%)	0.531
Negative	15 (42.9%)	35 (36.8%)	0.531

Frequency and duration of antipsychotic medication for the treatment of CB and NPS

In a total of 35 individuals with antipsychotic medication, there were 22 individuals with CB and 13 with NPS; the mean age of individuals with CB was 52.9 years, while the mean age of individuals with NPS was 49.9 years (Table 2). Most individuals (60.6% of individuals with CB and 73.3% of individuals with NPS) were prescribed second-generation antipsychotics. Olanzapine was more frequently administered for the prevention of NPS (26.7%) than for the prevention of CB (6.1%) (p = 0.045). The duration of antipsychotic use was >3 years in the majority of cases, with no significant difference between individuals with CB and individuals with NPS.

**Table 2:** Frequency and duration of antipsychotics use for the treatment of CB and NPS CB: Challenging behavior; NPS: Neuropsychiatric symptom; ID: Intellectual disability; M/F: Male/female; SD: Standard deviation.

	CB	NPS	P-value
Number of patients (M/F) (%)	22 (12/10) (62.9%)	13 (6/7) (37.1%)	0.631
Mean age, years (SD); range	52.9 (11.3); 32-72	49.0 (12.9); 19-63	0.386
*Antipsychotics frequency (%)*			
Second-generation antipsychotics	20 (60.6%)	11 (73.3%)	0.392
Risperidone	8 (24.2%)	4 (26.7%)	0.857
Olanzapine	2 (6.1%)	4 (26.7%)	0.045
Quetiapine	6 (18.2%)	3 (20.0%)	0.881
Aripiprazole	4 (12.1%)	0 (0.0%)	0.159
First-generation antipsychotics	13 (39.4%)	4 (26.7%)	0.392
Haloperidole	2 (6.1%)	1 (6.7%)	0.935
Fluphenazine	1 (3.0%)	0 (0.0%)	0.495
Chlorpromazine	1 (3.0%)	0 (0.0%)	0.495
Levomepromazine	6 (18.2%)	1 (6.7%)	0.294
Propericiazine	2 (6.1%)	0 (0.0%)	0.33
Sulpiride	1 (3.0%)	2 (13.3%)	0,171
Total number	33	15	
*Duration of antipsychotics use (%)*			
<3 years	3 (13.6%)	2 (15.4%)	0.886
≥3 years	19 (86.4%)	11 (84.6%)	0.886

Comparison of cardiometabolic parameters between ID with and without antipsychotic medication

The cardiometabolic effects of antipsychotic medication were analyzed by comparing the differences in metabolic parameters between IDs with and without antipsychotic medication (Table 3). The mean values of metabolic parameters (including BMI, blood pressure, serum lipid levels such as total cholesterol, high-density lipoprotein cholesterol, triglycerides, and QTc of ECG) were within the normal limits, and the individuals managed with and without antipsychotic medication did not differ to a statistically significant extent. However, the total calorie intake (p = 0.004) and the corrected calorie intake per BW kg (p = 0.042) were significantly higher in ID patients with antipsychotic medication than in those without antipsychotic medication.

**Table 3:** Comparison of cardiometabolic parameters between ID with and without antipsychotic medication ID: Intellectual disability; N: Number of patients; M/F: Male/female; SD, Standard deviation; BW: Body weight; BMI: Body mass index; QTc: Corrected QT interval. * All individuals are challenging behavior (CB) and neuropsychiatric symptom (NPS)-positive. ** All individuals are CB and NPS-negative.

	ID with antipsychotics medication (*) (N = 35)	ID without antipsychotics medication (**) (N = 95)	P-value
BW (kg), mean (SD); range	45.5 (12.4); 19.8-82.8	41.9 (8.5); 20.6-65.7	0.116
*Calorie intake per day*			
Total intake (kcal), mean (SD); range	1497 (460); 800-2500	1241 (334); 700-2100	0.004
Corrected intake (kcal/BW kg), mean (SD); range	34.3 (10.9); 16.8-57.1	30.1 (7.8); 15.4-50.6	0.042
BMI (kg/m^2^), mean (SD); range	19.2 (3.8); 13.7-35.8	18.5 (2.7); 11.6-24.6	0.238
*Blood pressure*			
Systole (mmHg), mean (SD); range	112 (16.0); 90-162	112 (12.7); 81-140	0.988
Diastole (mmHg), mean (SD); range	67 (12.1); 46-101	71 (11.5); 46-97	0.121
*Lipid*			
Total cholesterol (mg/dl), mean (SD); range	158.7 (30.7); 94-238	168.8 (34.8); 104-302	0.114
High-density lipoprotein cholesterol (mg/dl), mean (SD); range	53.2 (13.2); 33-87	54.6 (14.5); 32-91	0.61
Triglyceride (mg/dl), mean (SD); range	91.9 (40.4); 31-197	100.7 (58.6); 33-385	0.335
QTc (msec), mean (SD); range	418.1 (24.7); 356-498	416.2 (23.1); 322-478	0.692

Subscale prevalence of CB and NPS

**Figure 1:**
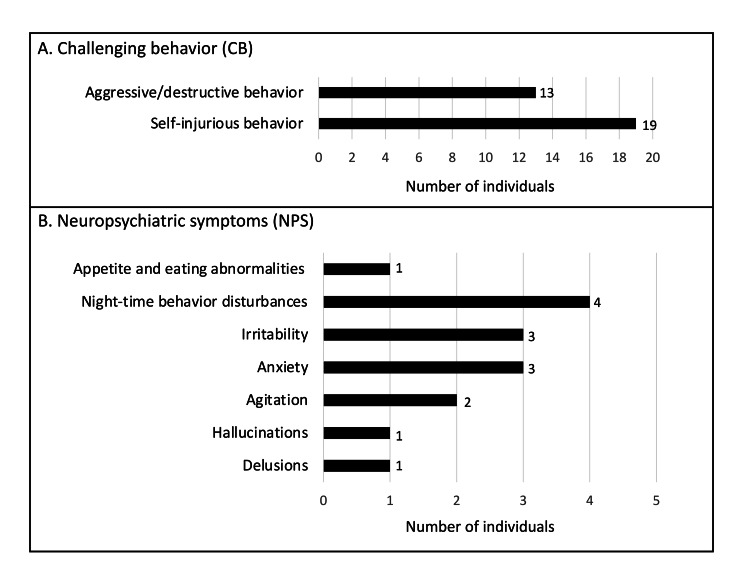
Number of individuals with CB and NPS Individuals with stereotyped behavior in challenging behavior (CB) and those exhibiting aberrant motor behavior, disinhibition, euphoria, apathy, and dysphoria in neuropsychiatric symptoms (NPS) were not observed.

The number of individuals with each subscale of CB and NPS is summarized in Figure 1. CB included 19 cases of self-injurious behavior and 13 cases of aggressive/destructive behavior. Individuals with stereotyped behavior were not observed. In NPS, night-time behavior disturbances were the most prevalent (n = 4) cases, followed by anxiety (n = 3), irritability (n = 3), and agitation (n = 2). Other symptoms were observed in one case or less.

## Discussion

Pathophysiological basis of behavioral disorder in autism spectrum disorder (ASD)

Challenging behaviors are frequent disorders among individuals with ASD, with an estimated prevalence of 56%-94% [10]. The pathogenetic mechanisms of challenging behaviors are not fully understood; however, it has been shown that a high proportion of children with ASD who manifested severe speech impairments used challenging behavior as a form of expressive communication [11], which could also increase when the environment is not congruent with the individual’s specific needs [12] and when sensory overstimulation or understimulation is produced [13]. It has also been indicated that the appearance of challenging behaviors is related to core symptom severity, levels of cognitive and language impairments, and medical comorbidity in individuals with ASD [14].

Medication is a valuable adjunct in the treatment of the behavioral symptoms of ASD and may help increase the efficacy of psychosocial interventions [15]. Risperidone is the first atypical antipsychotic to be approved by the United States Food and Drug Administration (FDA) for treating irritability in children and adolescents with autism. Second-generation or atypical antipsychotics differ from conventional antipsychotics in that they usually exhibit antagonism of serotonin (5-hydroxytryptamine [5-HT]) type 2A receptors in addition to blocking dopamine (DA) receptors. Several published reports have suggested the clinical effectiveness of risperidone, and a randomized controlled trial demonstrated the effectiveness of risperidone in the short-term treatment of symptoms of autism in adults [16]. Aripiprazole is also an atypical antipsychotic with potent partial agonism at dopamine D2 receptors in addition to properties as a 5-HT_1A _agonist and 5-HT_2_ antagonist [17]. Aripiprazole is approved in the United States for irritability associated with autism in children, and there is limited evidence in adults. However, some studies have reported the efficacy of aripiprazole in the treatment of challenging behaviors in adults with ASD [18]. This clinical evidence implies that the dysregulation of DA and 5-HT neurotransmission underlies the pathophysiology of behavioral disorders in ASD.

Mechanism of the cardiometabolic side effects of antipsychotics

Second-generation antipsychotics have become popular for the treatment of more than just psychotic disorders and are even used off-label for the treatment of behavioral disorders in ID [19]. These agents are associated with a substantial risk of metabolic complications, such as weight gain, dyslipidemia, insulin resistance, hypertension, and cardiac toxicity [20]. Regarding the mechanisms of cardiometabolic side effects, multiple studies have been reported.

Weight Gain

The hypothalamus consists of anatomically distinct nuclei that function as master regulators of appetite through signaling mediated by neuromodulators, including anorexigenic neuropeptides proopiomelanocortin (POMC) and orexigenic neuropeptide Y (NPY). POMC and NPY neurons contain serotonin receptors, and antagonization of 5-HT_2C_ and 5-HT_1/2A_ receptors induces NPY upregulation and POMC downregulation, which results in antipsychotic-induced hyperphagia and weight gain [21].

Dyslipidemia

Lipid abnormalities may follow observable weight gain associated with antipsychotic medications, which are thought to be caused by increased lipid biosynthesis through the induced gene expression of specific enzymes necessary for lipid metabolism [22]. Some antipsychotics are amphiphilic and act on cholesterol biosynthesis at the cellular level, namely, clozapine inhibits Δ−24 reductase and/or Δ−8,7 isomerase reactions, and risperidone inhibits Δ−7 reductase, Δ−14 reductase, and 14-α-demethylase [23]. This results in the accumulation of different sterol intermediates in the cholesterol biosynthesis pathway, leading to the increased synthesis of complex lipids (phospholipids and triglycerides) [24]*.*

Insulin Resistance

The onset of insulin resistance is heralded by postprandial hyperinsulinemia, which is followed by fasting hyperinsulinemia and ultimately by hyperglycemia [25]. Atypical antipsychotics have been found to decrease global DNA methylation, and associations between antipsychotic-induced insulin resistance and hypomethylation have been demonstrated [26]. A differentially methylated CpG site on the fatty acyl CoA reductase 2 (FAR2) gene of chromosome 12 has been reported to be associated with antipsychotic-induced insulin resistance in subjects with bipolar disorder [27].

Hypertension

The relationship between insulin resistance and hypertension is well established. Under normal physiologic conditions, insulin is a vasodilator with secondary effects on sodium reabsorption in the kidney. However, in the setting of insulin resistance, the vasodilatory effect of insulin is lost, while the renal effect on sodium reabsorption is preserved. Increased sodium reabsorption has the potential to increase arterial pressure, causing hypertension [25]. Aripiprazole is a 5-HT_2A_ receptor partial agonist. As 5-HT_2A_ causes contractions in the smooth muscles of the vasculature, aripiprazole may increase vessel resistance, resulting in hypertension [6].

Cardiotoxicity

Antipsychotic cardiotoxicity ranges from heart rate (HR) changes and blood pressure (BP) alterations to more severe and fatal issues, such as QTc prolongation and congestive heart failure [28]. Antipsychotic agents are commonly correlated with repolarization abnormalities, which manifest as iatrogenic prolongation of the QT interval. The QT interval is measured from the beginning of the QRS complex to the end of the T wave, which reflects depolarization and repolarization of the ventricles, respectively. An imbalance in ion flow across the cell membrane, especially potassium current impairment, can result in delayed repolarization manifesting a prolonged QT interval. It has been reported that some second-generation antipsychotics, including quetiapine and risperidone, are associated with prolongation of the QTc interval [29].

Weight control as the primary step against cardiometabolic adverse effects of antipsychotics

Metabolic complications from weight gain to cardiac toxicity by antipsychotics may appear consecutively as interrelated metabolic disorders. Therefore, monitoring for cardiometabolic side effects is necessary for any patient taking antipsychotic medication. First, increased appetite and weight gain can lead to elevated BMI and ultimately obesity. Thus, weight and BMI should be monitored. Second, some agents can cause insulin resistance by an unknown mechanism; this can be detected by measuring fasting plasma triglyceride levels. Finally, hyperinsulinemia may advance to pancreatic β-cell failure, prediabetes, and then diabetes. Diabetes increases the risk of cardiovascular events and premature death [30]. Total energy expenditure (TEE) comprises resting energy expenditure (REE, ~24 kcal/kg normal adult body weight/day), activity energy expenditure (~12 kcal/kg in healthy sedentary individuals), and the thermic effect of food (10% of TEE). The TEE of a healthy adult is ~36 kcal/kg [25]. REE can be measured by indirect calorimetry or estimated using a variety of predictive equations that include weight, height, age, sex, and sometimes disease-related factors as variables. The amount of energy they actually require may be less than TEE (as in obesity therapy) or greater than TEE (when rehabilitating nutritionally depleted patients) [25].

Limitations

This report is primarily a retrospective evaluation study and is not an interventional study. Therefore, several limitations are included in the methodology such as (1) the inclusion and exclusion criteria of participants, (2) the reliability and validity of the instruments for CB and NPS, and (3) the efficacy of antipsychotic use to the participants.

## Conclusions

This report examined the impact of long-term and off-label antipsychotic medication in cases with CB and NPS and demonstrated that the mean values of metabolic parameters were all normal in both medicated and nonmedicated individuals, while the total calorie intake was significantly higher in individuals treated with antipsychotic medication. We suggest that body weight control with adequate energy intake is the primary step in avoiding the cardiometabolic adverse effects of antipsychotics in intellectually disabled individuals with CB and NPS.
